# *Primulinajiulianshanensis*, a new species of Gesneriaceae from Jiangxi Province, China

**DOI:** 10.3897/phytokeys.226.96351

**Published:** 2023-05-09

**Authors:** Guo-Liang Xu, Li-Fen Liang, Di-Ya Chen, Zhi-Fang Jing, Xiao-Hai Zuo, Zheng-Yu Zuo, Fang Wen

**Affiliations:** 1 Jiulianshan National Nature Reserve Administrative Bureau, Longnan, CN-341700, China Jiulianshan National Nature Reserve Administrative Bureau Longnan China; 2 Jiangxi Environmental Engineering Vocational College, Ganzhou, CN-341000, China Jiangxi Environmental Engineering Vocational College Ganzhou China; 3 Guangxi Key Laboratory of Plant Conservation and Restoration Ecology in Karst Terrain, Guangxi Institute of Botany, Guangxi Zhuang Autonomous Region and Chinese Academy of Sciences, CN-541006, Guilin, China Gesneriad Committee of China Wild Plant Conservation Association Guilin China; 4 College of Tourism and Landscape Architecture, Guilin University of Technology, Guilin, CN-541006, China Guilin University of Technology Guilin China; 5 Germplasm Bank of Wild Species, Kunming Institute of Botany, Chinese Academy of Sciences, Kunming, CN-650201, China Germplasm Bank of Wild Species, Kunming Institute of Botany, Chinese Academy of Sciences Kunming China; 6 National Gesneriaceae Germplasm Resources Bank of GXIB, Gesneriad Committee of China Wild Plant Conservation Association, Gesneriad Conservation Center of China (GCCC), Guangxi Institute of Botany, Guilin Botanical Garden, Guangxi Zhuang Autonomous Region and Chinese Academy of Sciences, CN-541006, Guilin, China Guangxi Institute of Botany, Chinese Academy of Sciences Guilin China

**Keywords:** Flora of Jiangxi, Jiulianshan National Nature Reserve, new taxon, *Primulinawenii*, taxonomy

## Abstract

*Primulinajiulianshanensis* F.Wen & G.L.Xu, a new species of Gesneriaceae from Jiulianshan National Nature Reserve of Jiangxi Province, China, is described and illustrated here. Molecular evidence showed it was sister to *P.wenii* Jian Li & L.J.Yan, while the morphological observation found clear differences between them, petiole, both sides of leaf blades, adaxial surface of the calyx lobes, corolla inside toward the bottom, bract margins covered glandular-pubescent hairs in *P.jiulianshanensis* (*vs.* no glandular-pubescent hairs in *P.wenii*); lateral bracts 4–9 × ca. 2 mm, the central one 2–5 × 1–1.5 mm, adaxially glabrous but sparsely pubescent at apex (*vs.* lateral bracts 14–16 × 2.5–3.0 mm, the central one 10–12 × 1.3–1.6 mm, all adaxially pubescent); calyx lobes 8–11 × ca. 2 mm, each side with several brown serrate teeth at apex (*vs.* 14–15 × ca. 2.5 mm, margin entire); filaments and staminodes sparsely yellow glandular-puberulent (*vs.* white, glabrous).

## ﻿Introduction

The genus *Primulina* Hance (Hance 1883) in Gesneriaceae is mainly distributed in the mountainous areas of southern and southwestern China to northern Vietnam, especially in Karst landforms (Wei 2018; Xu et al. 2020b). Since it was redefined (Wang et al. 2011; Weber et al. 2011), many new species have been discovered and published. For example, ten new taxa of *Primulina* from China were reported in 2020 (Du et al. 2021). As of December 2022, about 240 species (including infraspecies, the same below) have been confirmed throughout the world, of which 224 species are distributed in China. So far, this genus is the largest genus of Gesneriaceae in China (POWO 2022).

Jiangxi Province is located in the mid-subtropical region of East China; the species number of *Primulina* is not very rich. After consulting references, checking herbarium specimens, and excluding the species that have been mistakenly identified, only 13 species of *Primulina* have been confirmed in Jiangxi Province (Peng et al. 2021). Three of them were discovered and published after 2011, which are *P.lepingensis* Z.L.Ning & M.Kang (Ning et al. 2014), *P.suichuanensis* X.L.Yu & J.J.Zhou (Zhou et al. 2016) and *P.inflata* Li H.Yang & M.Z.Xu (Xu et al. 2020a).

In April 2021, an interesting population of *Primulina* was found on a cliff of Danxia landform under the evergreen broad-leaved forest in Jiulianshan National Nature Reserve, Longnan City, Jiangxi Province. Morphologically, this species is similar to *P.wenii* Jian Li & L.J.Yan (Li et al. 2017) in some characteristics. For example, leaf blades are oblong or broadly rounded, corolla purple, and so on.

We collected the plants at the flowering and fruiting stage in the type locality to make specimens, and at the same time carried out botanical fine anatomical photography to observe carefully. We saw that the indumentum characters of petiole, leaf, bract, calyx, corolla tube, filaments, staminodes of this unknown species were obviously different from *P.wenii*, and the differences of two species’ calyx lobes and bracts characters can help us easily distinguish them. In order to understand the phylogenetic placements of this unknown species in *Primulina*, ITS and *trnL-F* sequences of this species were amplified and included for phylogenetic analysis to examine the relationships of the putative new species.

## ﻿Materials and methods

### ﻿Morphological observation

All available specimens of *Primulina* were used and compared (i. e. those stored in the following herbaria ANU, HITBC, IBK, IBSC, KUN, PE), as was the material of *Primulina* from recent fieldwork by the authors’ team in South and Southwest China. All the morphological characters, such as leaves, inflorescences, flowers and capsules, were observed and measured in the field. The description, measurements, shape, color and other details given in this description are based on living plants and specimens. We examined distinguished morphological characters of the presumed new species and the compared one, *P.wenii*, under a dissecting microscope. We described this presumed new species using the terminology of Wang et al. (1990, 1998).

### ﻿Sampling and DNA sequencing

We randomly selected one plant from the population to collect its leaves for a DNA experiment. Fresh leaf materials were preserved in silica gel for quick drying. Total genomic DNA was extracted from dried leaves using modified cetyltrimethylammonium bromide (CTAB) protocol (Doyle and Doyle 1987). ITS and *trnL-F* were amplified and sequenced following the methods of Möller et al. (2009) and Smissen et al. (2004), respectively. Besides, we downloaded the ITS and *trnL-F* sequences from GenBank for 188 *Primulina* species and two *Petrocodon* species. Species and GenBank accession numbers employed in this study are listed in Table 1.

**Table 1.** Species names and GenBank accession numbers of ITS and *trnL-F* DNA sequences used in this study.

Species name	Voucher number	ITS	*trnL-F*
*Petrocodonainsliifolius*	CWH88	KF202291	KF202298
*Petrocodonhancei*	CIPeng22903	KY796057	KY796059
*Primulinaalutacea*	YD07	KY394847	KY393441
*Primulinaargentea*	YMBC	KY394848	KY393442
*Primulinabaishouensis*	GXLG05	KY394849	KY393443
*Primulinabalansae*	BALAN	MK747141	MK746274
*Primulinabeiliuensis*	GXBLBC	KY394850	KY393444
Primulinabeiliuensisvar.fimbribracteata	SGQJ04	KY394851	KY393445
*Primulinabicolor*	SLHLCB	KY394852	KY393446
*Primulinabipinnatifida*	GXLG04	KY394853	KY393447
*Primulinabobaiensis*	BBGL01	KY394854	KY393448
*Primulinabogneriana*	WF7	MK747166	MK746225
*Primulinabrachytricha*	DWDMCZ	KF498048	KY393450
Primulinabrachytrichavar.magnibracteata	KFC4193	MK369979	MK369994
*Primulinabrunnea*	BRUN	MK747142	MK746275
*Primulinabullata*	GXJX06	KF498071	KY393451
*Primulinacangwuensis*	GXLG04	KY394853	KY393447
*Primulinacardaminifolia*	GXLB	MK747131	MK746255
*Primulinacarinata*	NTBC	KY394858	KY393452
*Primulinacataractarum*	N1	MW900263	MW960358
*Primulinachizhouensis*	JXFY01	KY394860	KY393454
*Primulinacolaniae*	WF8	MK747167	MK746224
*Primulinaconfertiflora*	GDYS05	MK747101	MK746253
*Primulinacordata*	HYH010	KC190200	KC190207
*Primulinacordifolia*	GXRA02	KY394863	KY393457
*Primulinacordistigma*	GDYCXZ	MK747118	MK746251
*Primulinacrassirhizoma*	CJGL01	KY394864	KY393458
*Primulinacrassituba*	HNSP	MK747147	MK746230
*Primulinacurvituba*	GXHJ01	MK747137	MK746242
*Primulinadanxiaensis*	P22865	JX506886	JX506778
*Primulinadebaoensis*	DBGL01	KY394868	KY393462
*Primulinadepressa*	DXS02	KY394869	KY393463
*Primulinadiffusa*	PJGL01	KY394871	KY393465
*Primulinadongguanica*	DGBC	KY394872	KY393466
*Primulinadrakei*	YNCP01	KY394873	KY393467
*Primulinadryas*	HKDMS	KY394875	KY393469
*Primulinaduanensis*	DABC	KY394877	KY393471
*Primulinaeburnea*	P22908	JX506891	JX506783
*Primulinaeffusa*	KFC4167	MK369976	MK369991
*Primulinafengkaiensis*	KFC4130	MK369975	MK369990
*Primulinafengshanensis*	KFC4195	MK369970	MK369985
*Primulinafimbrisepala*	P22863	JX506894	JX506786
Primulinafimbrisepalavar.mollis	GXIB	JX506895	JX506787
*Primulinaflavimaculata*	KFC3988	MK369974	MK369989
*Primulinafloribunda*	DHGL01	KY394886	KY393480
*Primulinafordii*	LJM1207202	MG727881	MG727878
Primulinafordiivar.dolichotricha	DHS01	MK747125	MK746247
*Primulinagemella*	GEME	MK747146	MK746254
*Primulinaglabrescens*	GZLBSM	MK747132	MK746278
*Primulinaglandaceistriata*	GXLCHW	MK747114	MK746256
*Primulinaglandulosa*	GXPLCG	KY394887	KY393481
*Primulinagongchengensis*	GCGL01	KY394889	KY393483
*Primulinagrandibracteata*	YNHK	MK747121	MK746266
*Primulinaguigangensis*	GXGGBC	KY394892	KY393486
*Primulinaguihaiensis*	GXLG036	KY394893	KY393487
*Primulinaguizhongensis*	GXGZBC	KY394894	KY393488
*Primulinahalongensis*	HLW01	KY394895	KY393489
*Primulinahedyotidea*	XWB	JX506905	JX506797
*Primulinaheterochroa*	GXMES01	KY394898	KY393492
*Primulinaheterotricha*	HNBT01	KY394899	KY393493
*Primulinahezhouensis*	HZXH	MK747143	MK746258
*Primulinahiepii*	WF2	MK747144	MK746223
*Primulinahochiensis*	GXIB	JX506903	JX506795
*Primulinahuaijiensis*	GDHJ02	KF498127	KY393495
*Primulinahuangii*	WF12	MK747138	MK746231
*Primulinahunanensis*	Xu11697	KU220602	KU220608
*Primulinajiangyongensis*	HNJY01	KY394902	KY393496
*Primulinajingxiensis*	LZXHGL01	KY394903	KY393497
*Primulinajiuwanshanica*	JWS	MK747116	MK746260
*Primulinajuliae*	LJM1210011	MG727889	MG727873
*Primulinalangshanica*	LSCZ	KY394907	KY393501
*Primulinalatinervis*	XIN1	KY394908	KY393502
*Primulinalaxiflora*	P22927	JX506910	JX506802
*Primulinalechangensis*	GDLC12	KY394910	KY393504
*Primulinaleeii*	LSGL01	KY394911	KY393505
*Primulinaleiophylla*	GXJX07	KY394912	KY393506
*Primulinalepingensis*	JXLP01	KY394913	KY394913
*Primulinaleprosa*	GXMS055	KY394914	KY393508
*Primulinalianpingensis*	CHLT016	MH343910	MH344542
*Primulinaliboensis*	GXJX08	KY394917	KY393511
*Primulinaliguliformis*	GXIB	JX506912	JX506804
*Primulinalijiangensis*	GLS01	KY394919	KY393513
*Primulinalinearicalyx*	KFC4141	MH032854	MH032841
*Primulinalinearifolia*	GXNN01	KY394921	KY393515
*Primulinalingchuanensis*	LCXHGL01	KY394922	KY393516
*Primulinalinglingensis*	LLBC	KY394923	KY393517
Primulinalinglingensisvar.fragrans	XHLLBC2	MK746285	MK746285
*Primulinaliujiangensis*	LJGL01	KY394924	KY393518
*Primulinalobulata*	GDQX04	KF498054	KY393519
*Primulinalonggangensis*	P22948	JX506916	JX506808
*Primulinalongicalyx*	GXGL01	KY394927	KY393521
*Primulinalongii*	XWB	JX506917	JX506809
*Primulinalongzhouensis*	P22963	JX506918	JX506810
*Primulinalunglinensis*	GZXY04	KY394930	KY393524
Primulinalunglinensisvar.amblyosepala	LCDE	MK747105	MK746281
*Primulinalungzhouensis*	GXJX10	KY394931	KY393525
*Primulinaluochengensis*	LCWCGL01	KY394932	KY393526
*Primulinalutea*	1844	JX506921	JX506813
*Primulinalutescens*	PBLS01	MK747135	MK746263
*Primulinalutvittata*	KFC4149	MK369978	MK369993
*Primulinaluzhaiensis*	HYH019	KC190197	KC190204
*Primulinamabaensis*	SZY02	KY394937	KY393531
*Primulinamacrodonta*	GXIB	JX506923	JX506815
*Primulinamaculata*	Xu11916	KU220604	KU220609
*Primulinamaguanensis*	YNMG	MK747127	MK746267
*Primulinamalipoensis*	YNMLP01	MK747123	MK746240
*Primulinamedica*	GXPLCM	KY394940	KY393534
*Primulinamelanofilamenta*	GXXA	MK747158	MK746277
*Primulinaminor*	WXXH1	MK747160	MK746290
*Primulinaminutimaculata*	GXLZ10	KY394941	KY393535
*Primulinamoi*	SGWY03	KF498115	KY393536
*Primulinamollifolia*	GXESWC	KY394943	KY393537
*Primulinamultifida*	DLXHGL01	KY394946	KY393540
*Primulinanandanensis*	GXJX02	KY393541	KY393541
*Primulinanapoensis*	GXIB	JX506930	JX506821
*Primulinaningmingensis*	NMGL01	KY394949	KY393543
*Primulinaobtusidentata*	GZJK01	KF498096	KY393544
*Primulinaophiopogoides*	GXFS01	KF498062	KY393545
*Primulinaorthandra*	ZRBC2	MK747128	MK746286
*Primulinaparvifolia*	GGSL01	KY394952	KY393546
*Primulinapengii*	W0397	KU220603	KU220610
*Primulinapetrocosmeoides*	SHDBC	KY394953	KY393547
*Primulinapinnatifida*	MS02	KY394954	KY393548
*Primulinapolycephala*	GDLZ06	KY394955	KY393549
*Primulinaporphyrea*	DNGL01	KU173793	KU173799
*Primulinapseudoeburnea*	KY394958	KY394958	KY393552
*Primulinapseudoglandulosa*	GXYS06	KF498138	KY393482
*Primulinapseudoheterotricha*	XWB	JX506933	JX506824
*Primulinapseudolinearifolia*	JXY	MK747140	MK746280
*Primulinapseudomollifolia*	JMMXH1	MK747134	MK746244
*Primulinapseudoroseoalba*	JFHGL01	KY394959	KY393553
*Primulinapteropoda*	HNCJ01	KY394960	KY393554
*Primulinapungentisepala*	JEGL01	KY394962	KY393556
*Primulinapurpurea*	ZHGL01	KY394964	KY393558
*Primulinaqingyuanensis*	GDQX01	KY394965	KY394965
*Primulinarenifolia*	GXDA02	KY394966	KY393560
*Primulinarepanda*	GXBM03	KY394968	KY393562
*Primulinaronganensis*	GXRA01	KF498135	KY393564
*Primulinarongshuiensis*	GXRS01	KF498088	KY393565
*Primulinaroseoalba*	LDGL01	KY394972	KY393566
*Primulinarosulata*	GXPL05	KU528874	KU528884
*Primulinarotundifolia*	OO3	KY394975	KY393569
*Primulinarubribracteata*	JH01R	KU173791	KU173797
*Primulinasclerophylla*	GXDA01	KY394979	KY393573
*Primulinasecundiflora*	GZQZ	MK747119	MK746279
*Primulinashouchengensis*	GXYF02	KY394980	KY393574
*Primulinasichuanensis*	SCBC	MK747162	MK746264
*Primulinadryas*	HKDMS	KY394875	KY393469
*Primulinasinovietnamica*	Peng21956	MK369973	MK369988
*Primulinaspinulosa*	GXFS02	KF498063	KY393576
*Primulinasubrhomboidea*	GXYS02	KY395018	KY393577
*Primulinasubulata*	GDYA01	KY395020	KY393579
Primulinasubulatavar.guilinensis	GXHYXH	KY394967	KY393561
*Primulinasubulatisepala*	CQAYH01	MK747122	MK746246
*Primulinasuichuanensis*	GDLC07	KY395021	KY393580
*Primulinaswinglei*	GXRX01	KY395022	KY393581
*Primulinatabacum*	LZ01	KY395023	KY393582
*Primulinatenuifolia*	GXBM01	KY395024	KY393583
*Primulinatenuituba*	GZGY01	KY395025	KY393584
*Primulinatiandengensis*	GXTD03	KY395027	KY393586
*Primulinatribracteata*	GXFS04	KY395028	KY393587
Primulinatribracteatavar.zhuana	1877	JX506952	JX506843
*Primulinatsoongii*	ZSGL01	KY395029	KY393588
*Primulinavaricolor*	GXNP01	KF498086	KY393589
*Primulinaverecunda*	LBJX01	KY395031	KY393590
*Primulinaversicolor*	GDYD01	MK747155	MK746252
*Primulinavestita*	QZXT	MK747156	MK746282
*Primulinavillosissima*	QXY01	KY395032	KY393591
*Primulinawenii*	WENI	MK747148	MK746284
*Primulinawentsaii*	GXLZ047	KY395033	KY393592
*Primulinawuae*	WSBC	MK747159	MK746265
*Primulinaxinningensis*	GGGL01	KY394891	KY393485
*Primulinaxiziae*	ZJHZ01	KY395038	KY393597
*Primulinayangchunensis*	GDYC01	KY395039	KY393598
*Primulinayangshanensis*	GDNX01	KY395040	KY393599
*Primulinayangshuoensis*	GXYS07	KY395042	KY393601
*Primulinayingdeensis*	YD03	KU528876	KU528886
*Primulinayungfuensis*	GXIB	JX506957	JX506848
*Primulinazhoui*	WF18	MK747104	MK746222
*Primulinajiulianshanensis*	WF217	OP243287	OP243283

### ﻿Phylogenetic analysis

We assembled and aligned the newly obtained sequences and those from GenBank using MAFFT v.7.017 (Katoh et al. 2002) and subsequently corrected and combined the ITS and *trnL-F* sequences in Geneious 9.1.4 (Kearse et al. 2012). We used the Maximum likelihood (ML) and Bayesian inference (BI) analyses to do the phylogenetic analysis of the ITS and *trnL-F* matrixes, and the combined ITS + *trnL-F* sequences data-set. The two best supported tree topologies from maximum likelihood (ML) analyses of ITS and *trnL-F* were visually compared for topological incongruence. A conflict in tree topologies of each tree was considered significant when incongruent topologies both received bootstrap values ≥ 80% (Fu et al. 2022). The ML analyses were conducted using IQ-TREE 1.6.12 (Nguyen et al. 2015) with the GTR+R6 model and 1000 ultrafast bootstrap replicates. For BI analysis, we employed MrBayes v.3.2.6 (Ronquist et al. 2012) to obtain a maximum clade credibility (MCC) tree. Bayesian inference was performed using one million generations, four runs, four chains, a temperature of 0.001, 25% trees discarded as burn-in, and trees sampled every 1,000 generations (1,000 trees sampled in total) with GTR+I+G model.

## ﻿Results

### ﻿Phylogenetic analysis

**Figure 1.**
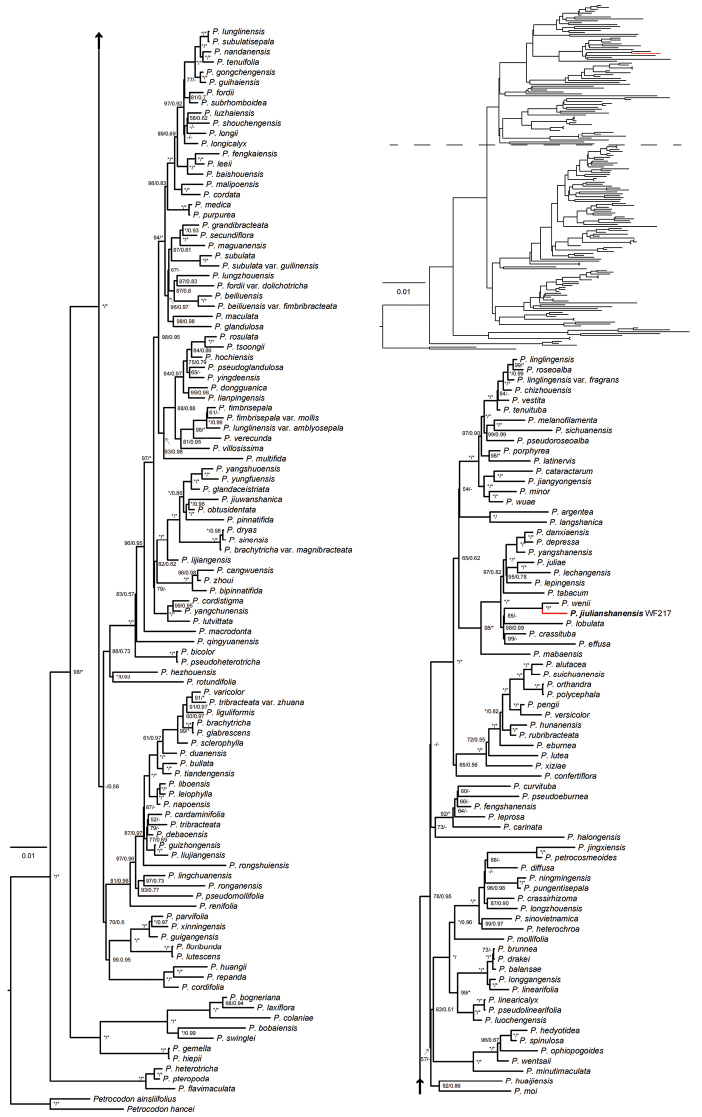
Phylogenetic tree of *Primulina* generated from maximum likelihood (ML) of combined *trn*L-F and ITS data-set. Numbers of each branch are support values in the order of UFBoot/BIPP. Stars indicate UFBoot = 100% or BIPP = 1.0. The dash (–) indicates a node with UFBoot or < 50% or BIPP < 0.5.

The ITS matrix had a length of 782 characters, with 449 (57.4%) variable characters and 355 (45.4%) parsimony-informative. In comparison, the *trnL-F* matrix had a length of 836 characters, with 198 (23.6%) variable characters and 93 (11.1%) were parsimony-informative. The comparison of trees for ITS and *trnL-F* revealed no significant incongruence topology and both indicated that *Primulinajiuyishanensis* is closely related to *P.wenii* (Suppl. materials 1, 2). Because the combined dataset resulted in a better-resolved tree with higher support values, we use the combined dataset to do the further molecular studies. The combined data-set was 1628 characters, with 653 (40.1%) variable characters and 452 (27.8%) parsimony-informative, including the indels in all matrixes. The undescribed species and *P.wenii* were sister groups [Bayesian posterior probabilities (BIPP) = 1.00, ML ultrafast bootstrap support values (UFBoot) = 100%], and we found 10 and 1 different sites in the ITS (totally 615 bp) and the *trnL-F* (totally 750 bp) sequences between them, respectively (Table 2). belonging to a strongly supported clade (BIPP = 0.99, UFBoot = 98%) included *Primulinacrassituba* (W.T.Wang) Mich.Möller & A.Weber, *P.effusa* F.Wen & B.Pan and *P.lobulata* (W.T.Wang) Mich.Möller & A.Weber (Wang 1982, 1989; Weber et al. 2011; Pan et al. 2017) (Fig. 1).

**Table 2.** Sequence differences of ITS and *trnL-F* regions between *Primulinajiulianshanensis* and *P.wenii*.

Species & marker	Sequences
*P.jiulianshanensis* ITS	1~CCCGAGAACATGTTTAAAACACGCTTGCGT~30
*P.wenii* ITS	1~CCCGAGAACATGTTTAAGACACGCTTGCGT~30
*P.jiulianshanensis* ITS	141~CGAGCGCCTCTCCGTCCTGGCTAAGTTCGC~170
*P.wenii* ITS	141~CGAGCGCCTCTCCGTACCGGTGAAGTTCGC~170
*P.jiulianshanensis* ITS	396~CGTTTTTTCCACGCTCAAAAGGTGTC–GGGGACGA~430
*P.wenii* ITS	396~CGTCTTTTCCACGCTCCAAAGGTGTCGGGGGAAGA~430
*P.jiulianshanensis trnL-F*	501~GTTCAAAAGTCCTTTATCTT~520
*P.wenii* *trnL-F*	501~GTTCAAAATTCCTTTATCTT~520

### ﻿Taxonomic treatment

#### ﻿ Primulina jiulianshanensis

Taxon classificationPlantaeLamialesGesneriaceae

F.Wen & G.L.Xu sp.nov.

F64C620E-DC3C-5C46-87B4-F1EC4700AAA7

urn:lsid:ipni.org:names:77318694-1

Figs 2 , 3

##### Diagnosis.

This new species differs from *P.wenii* by the combination of the following characteristics: petiole, both sides of leaf blades, adaxial surface of the calyx lobes, corolla inside toward the bottom, bract margins glandular-pubescent (*vs.* what above-mentioned eglandular-pubescent in *P.wenii*); lateral bracts 4–9 × ca. 2 mm, the central one 2–5 × 1–1.5 mm, adaxially glabrous but sparsely pubescent at apex (*vs.* lateral bracts 14–16 × 2.5–3.0 mm, the central one 10–12 × 1.3–1.6 mm, all adaxially pubescent); calyx lobes 8–11 × ca. 2 mm, each side with several brown serrate teeth at apex (*vs.* 14–15 × ca. 2.5 mm and entire); filaments and staminodes sparsely glandular-puberulent (*vs.* glabrous). Detailed morphological comparisons with *P.wenii* are provided in Table 3.

**Table 3.** Comparisons between the characters of *Primulinajiulianshanensis* and *P.wenii*.

Characters	*Primulinajiulianshanensis*	*P.wenii*
Petiole indumentum	densely villous and very sparsely glandular pubescent	densely villous
Leaf blades indumentum	adaxially densely white to light red villous and very sparsely glandular pubescent, abaxially densely white villous and pubescent, and very sparsely glandular -pubescent	adaxially densely pubescent and villous, abaxially densely appressed pubescent
Bract	lateral ones 4–9 × ca. 2 mm, the middle one 2–5 × 1–1.5 mm, all abaxially white or reddish pubescent, adaxially glabrous but sparsely pubescent at apex, margin ciliate and very sparsely glandular-puberulent	lateral ones 14–16 × 2.5–3.0 mm, the central one 10–12 × 1.3–1.6 mm; outside white pubescent and villous, adaxially white pubescent, margin entire and ciliate
Calyx lobes	adaxially sparsely white puberulent and glandular puberulent, margin entire but each side of calyx lobes with 1–3 brown crenate teeth at apex; lobes 8–11 × ca. 2 mm	adaxially sparsely shortly pubescent to nearly glabrous, margin entire, lobes 14–15 × ca. 2.5 mm
Filament	yellow from middle to base but white upper half, glandular-puberulent	white, glabrous
Staminodes	yellowish, lateral ones very sparsely glandular-puberulent	white, glabrous

**Figure 2.**
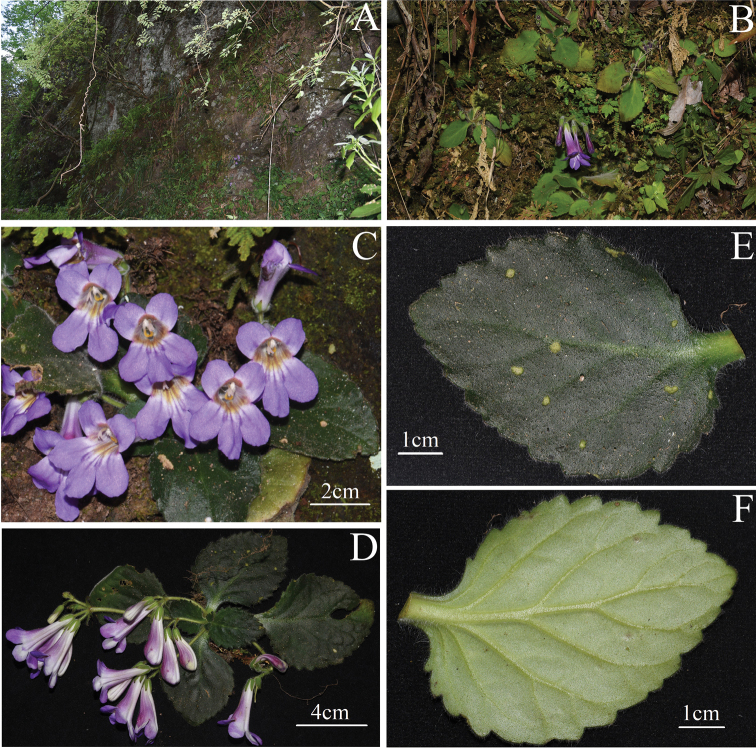
*Primulinajiulianshanensis* sp. nov. **A** habitat **B** population **C** plant in blooming **D** habit **E** adaxial surface of mature leaf blades and petiole **F** abaxial surface of mature leaf blade and petiole.

##### Holotype.

China, Jiangxi Province: Ganzhou City, Longnan County, Jiulianshan National Nature Reserve, growing in shady and moist cliffs in the forest, 24°34'8.9"N, 114°25'49.7"E, altitude ca. 440 m, April 20, 2021, *Guo-Liang Xu, JLSXGL-20210420* (Holotype IBK!; Isotype: KUN!)

**Figure 3.**
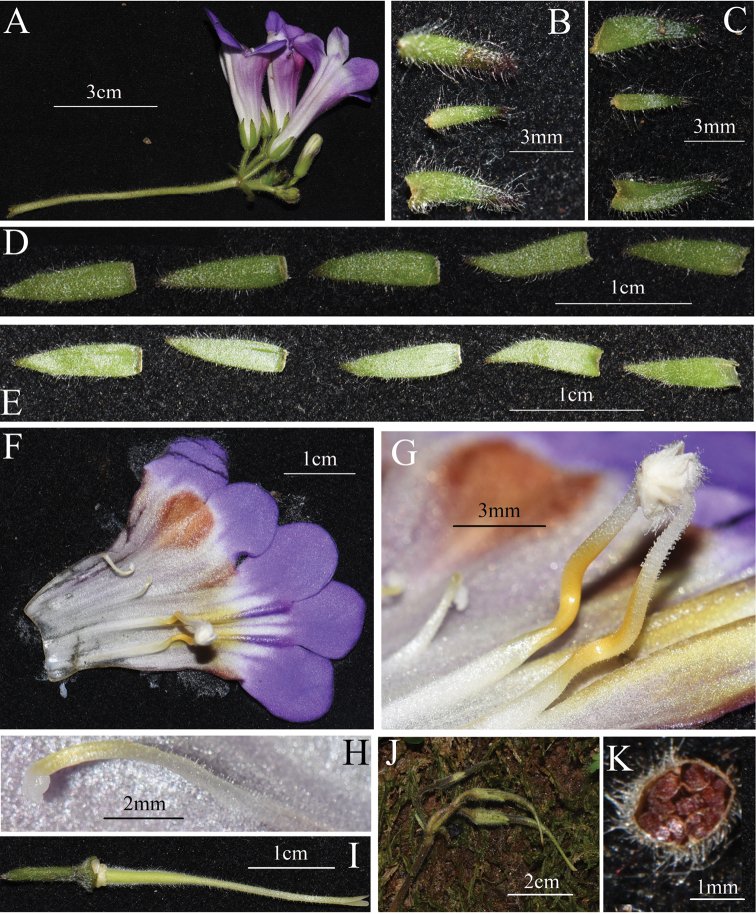
*Primulinajiulianshanensis* sp. nov. **A** cyme **B** adaxial surfaces of bracts **C** abaxial surfaces of bracts **D** adaxial surfaces of calyx lobes **E** abaxial surfaces of calyx lobes **F** opened corolla **G** stamens and anthers **H** one of lateral staminodes **I** pistil **J** immature capsules **K** transverse section of capsule.

##### Description.

Herbs perennial, acaulescent, rhizome terete, 1–4 cm long, 0.8–1.5 cm in diam., leaves all basal, 4–8, petiole 10–40 × 4–13 mm, densely villous and very sparsely glandular-pubescent. Leaf blade oblong-elliptic or broadly elliptic, 4–13 × 4–8 cm, thickly chartaceous, more and less fleshly, adaxially pale green to dark green, densely white to light red villous and very sparsely glandular-pubescent, abaxially pale green, densely white villous and pubescent, and very sparsely glandular-pubescent, base apparently asymmetric but cuneate, margin irregularly obtuse-serrate, apex slightly obtuse, lateral veins 4–6 on each side, adaxially inconspicuously sunken, abaxially prominently raised. Inflorescence dichotomous cymes 2–4, axillary, 1–3-branched, 3–9-flowered or more; peduncle and pedicle green, erectly white or light red pubescent; peduncle 5–10 cm long, 2–3 mm in diam.; pedicle 5–15 mm long, 1–2 mm in diam. Bracts 3, lanceolate or spatulate, a pair on either side in same size, opposite, 4–9 mm long, ca. 2 mm wide, the middle one smaller, 2–5 mm long, 1–1.5 mm wide, all three abaxially white or reddish pubescent, adaxially glabrous but sparsely pubescent at apex, margin entire, ciliate and very sparsely glandular-puberulent, apex acute; bracteoles 3, shape and indumentum same as bracts, 2–5 mm long, 1–1.5 mm wide. Calyx 5-parted, lobes lanceolate, 8–11 mm long, ca. 2 mm wide, nearly equal, abaxially densely white or light red villous, adaxially sparsely white puberulent and glandular-puberulent, margin entire but each side of calyx lobes with 1–3 purplish brown crenate at the apex. Corolla pinkish purple to bluish purple, 3.6–4.0 cm long; corolla tube funnelform, 2.6–3 cm long, mouth 1.3–1.6 cm in diam., base ca. 5 mm in diam., outside densely glandular-pubescent, inside from the middle to the base sparsely glandular-puberulent, and the upper part of the corolla tube glabrous; corolla tube abdomen with two obviously longitudinal ridges, the upper part (close to the mouth) of the longitudinal ridge dark bluish purple, and the lower part (close to the bottom) changing into yellowish brown; a dark reddish-brown lump on the upper throat of the corolla tube inside and between upper lip lobes, ovate to spatulate, extending to the middle of the corolla tube, the lump densely glandular-puberulent; a narrow triangular thickened dark reddish-brown stripe extending to the middle of the corolla tube inside at each side of corolla tube and at the junction of the abaxial and adaxial lip; limb distinctly 2-lipped, adaxial lip 2-parted to the middle, lobes broadly ovate to semicircular, apex round, 6–8 mm long, 6–9 mm wide at the bottom; abaxial lip 3-parted to near the base, lobes elliptical to oblong, 8–12 mm long, 7–9 mm wide at the bottom. Stamens 2, adnate to 1.8 cm above the base of corolla tube; filaments linear, yellow from middle to base but white upper half, 8–11 mm long, geniculate near the base, glandular-puberulent; anthers reniform, slightly constricted at the middle, densely villous and fewer glandular-puberulent; staminodes 3, yellowish, lateral ones ca. 6 mm long, adnate to 14 mm above the base of corolla tube, straight, linear, very sparsely glandular-puberulent, apex capitate, the central one ca. 1 mm long, adnate to 5–6 mm above the base of corolla tube, glabrous. Disc annular, ca. 1 mm high, margin undulate, glabrous, white. Pistil pale green, 2.8–3.2 cm long; style linear, 1.9–2.3 cm long, ca. 1 mm in diam., upper part densely glandular-puberulent, lower part densely glandular-puberulent and eglandular-puberulent, ovary oblong, ca. 10 mm long, ca. 2 mm in diam., densely villous and glandular-pubescent, parietal placenta. Stigma acute triangle to narrowly obtrapeziform, 2-lobed, ca. 3 mm long. Capsule linear, 2–4 cm long, parietal placenta, densely villous and glandular-pubescent.

##### Phenology.

Flowering from April to May, fruiting from June to September.

##### Etymology.

The specific epithet ‘*jiulianshanensis*’ is derived from the type locality, Jiulianshan National Natural Reserve, Jiangxi Province, China.

##### Vernacular name.

九连山报春苣苔 (Chinese name); Jǐu Lían Shān Bào Chūn Jù Tái (Chinese pronunciation).

##### Distribution and habitat.

We found three small subpopulations in Jiulianshan National Nature Reserve in Jiangxi Province, which are distributed in the shady and wet place on the cliffs under the evergreen broad-leaved forest in the reserve. And the new species is mainly acccompanied by *Begoniapalmata* D.Don, *Utriculariastriatula* J.Smith, *Selaginellamoellendorffii* Hieron., *S.involvens* (Sw.) Spring, etc.

##### Conservation status.

At present, only three small subpopulations with total ca. 300 mature individuals of the new species are known in the type locality, Jiulianshan National Natural Reserve, Jiangxi Province, China. The three subpopulations are stable because they are in the reserve. The known AOO and EOO of the new species are about 0.2 km^2^ and 25 m^2^, respectively. Thus, if considering its fewer individuals of three subpopulations, it should be temporarily assessed as Near Threated (NT), following the IUCN Red List Categories and Criteria (IUCN Standards and petitions committee 2022).

##### Notes.

The mainly morphological differences are showed in diagnosis and Table 3. In addition, the insides of the corolla tube are also somewhat different. For example, at the junction of the abaxial and adaxial lip, there are two narrow triangular thickened dark reddish-brown stripes inside the corolla tube in *Primulinajiulianshanensis*, but there are only two bluish purple spots at the same places in *P.wenii*; at corolla tube abdomen of *P.jiulianshanensis*, the upper parts of the longitudinal ridges are dark bluish purple, and the lower parts are yellowish brown, but the longitudinal ridges inside corolla tube of *P.wenii* are all dark bluish purple; the lump on the upper throat inside corolla tube of *P.jiulianshanensis* is dark reddish-brown, but *P.wenii* is dark bluish brown(Li et al. 2017).

The type locality of *Primulinawenii* is Rixi Township, Fuzhou, Fujian Province, while the type locality of *P.jiulianshanensis* is Jiulian Mountain, Jiangxi Province. Their type localities are separated by the Wuyi Mountains, and the two places are more than 500 kilometers apart. *P.wenii* has a narrow distribution range and is only recorded in Rixi Township, Fuzhou, Fujian Province (Li et al. 2017). This area is coastal and the climate zone of this region belongs to the typical subtropical marine monsoon climate. *P.wenii* grows in the limestone evergreen broad-leaved forest area with stable morphology. *P.jiulianshanensis*, grows on the rocks under the evergreen broad-leaved forest in Jiulianshan Nature Reserve, which is a typical Danxia landform. The soil forming rock are mainly sandstone and conglomerate in Jiulianshan Nature Reserve, which has a subtropical monsoon humid climate. The morphology of the *P.jiulianshanensis* in three subpopulations is also relatively stable. Therefore, considering the differences in molecular, morphological, and habitats between *P.jiulianshanensis* and *P.wenii*, they should be classified as two different species.

Except for a few species, such as *Primulinafimbrisepala* (Hand.-Mazz.) Yin Z.Wang, *P.eburnea* (Hance)Yin Z.Wang, *P.tenuituba* (W.T.Wang) Yin Z.Wang, *P.juliae* (Hance) Mich.Möller & A.Weber, most of the species of *Primulina* are narrowly distributed and endemic. Among the known species worldwide, more than 170 species are endemic to Karst areas in southern to southwestern China and to northern Vietnam (Wei 2018; Xu et al. 2020b). However, the diversity of *Primulina* in the Danxia landform has not been well understood so far (Yu et al. 2019). For example, the new species, *P.suichuanensis*, was found in the Danxia landform in Jiangxi Province (Zhou et al. 2016). It should be noted that a new provincial record for *Primulinawenii* from Jiangxi Province was discovered in Jiulianshan Nature Reserve (Liao et al. 2020). However, we carefully examined the voucher specimen (No. PVHJX-05557, stored in GNNU), and we noted that the voucher was collected from the same subpopulation of *P.jiulianshanensis* from the same site in Jiulianshan Nature Reserve. Further, we found there are many inconsistencies between the morphological description from the article (Liao et al. 2020) and the corresponding voucher specimens. Thus, this species’ new provincial record of *P.wenii* in Jiangxi province is a mistaken identification. Lastly, none of the close relatives of *Primulina* are morphologically similar to this new species found in Jiangxi (Fig. 1). Thus, the new taxon is not easily confused with the others in this province.

## Supplementary Material

XML Treatment for Primulina jiulianshanensis

## Supplementary materials

Supplementary material 1 The tree of the trnL-F plastid marker for *Primulina* species Data type (phylogenetic, genomic, tree) Explanation note The *trnL-F* plastid marker and the ITS nuclear marker separately and discuss any incongruences between the two markers, particularly with respect to the position of the new species. https://binary.pensoft.net/file/847546This dataset is made available under the Open Database License (http://opendatacommons.org/licenses/odbl/1.0/). The Open Database License (ODbL) is a license agreement intended to allow users to freely share, modify, and use this Dataset while maintaining this same freedom for others, provided that the original source and author(s) are credited.Author: Zheng-Yu Zuo

Supplementary material 2 The tree of the ITS nuclear marker for *Primulina* species Data type (phylogenetic, genomic, tree) Explanation note The *trnL-F* plastid marker and the ITS nuclear marker separately and discuss any incongruences between the two markers, particularly with respect to the position of the new species. https://binary.pensoft.net/file/847547This dataset is made available under the Open Database License (http://opendatacommons.org/licenses/odbl/1.0/). The Open Database License (ODbL) is a license agreement intended to allow users to freely share, modify, and use this Dataset while maintaining this same freedom for others, provided that the original source and author(s) are credited.Author: Zheng-Yu Zuo
