# Supplementary figures and images for: Primulinajiulianshanensis, a new species of Gesneriaceae from Jiangxi Province, China
Source: PhytoKeys. 2023 May 9;226:1–16. doi: 10.3897/phytokeys.226.96351 (PMC10189641; doi:10.3897/phytokeys.226.96351)

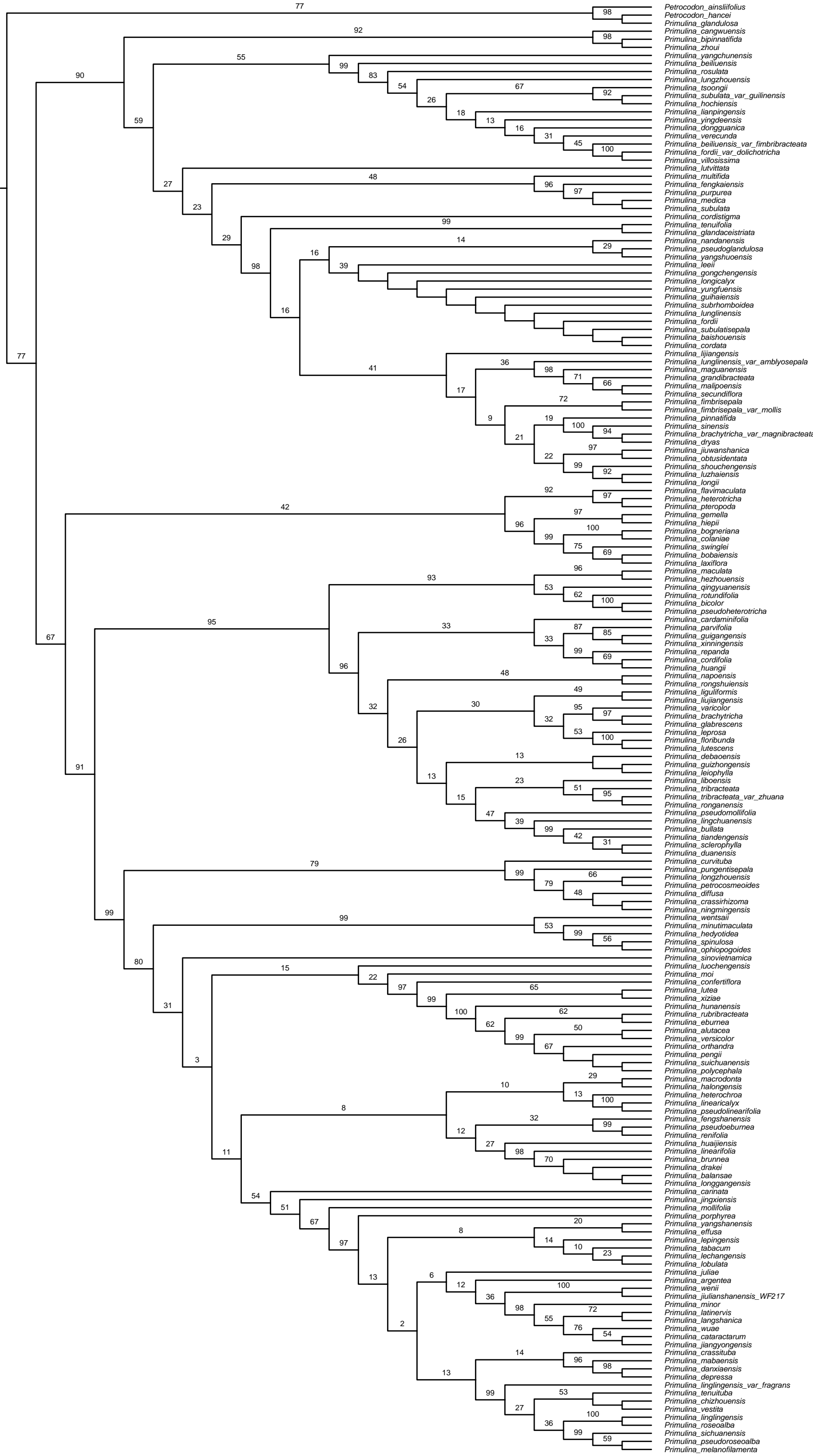

Supplement: Supplementary material 1 — The tree of the trnL-F plastid marker for Primulina species [file phytokeys-226-001_article-96351__-s001.pdf]

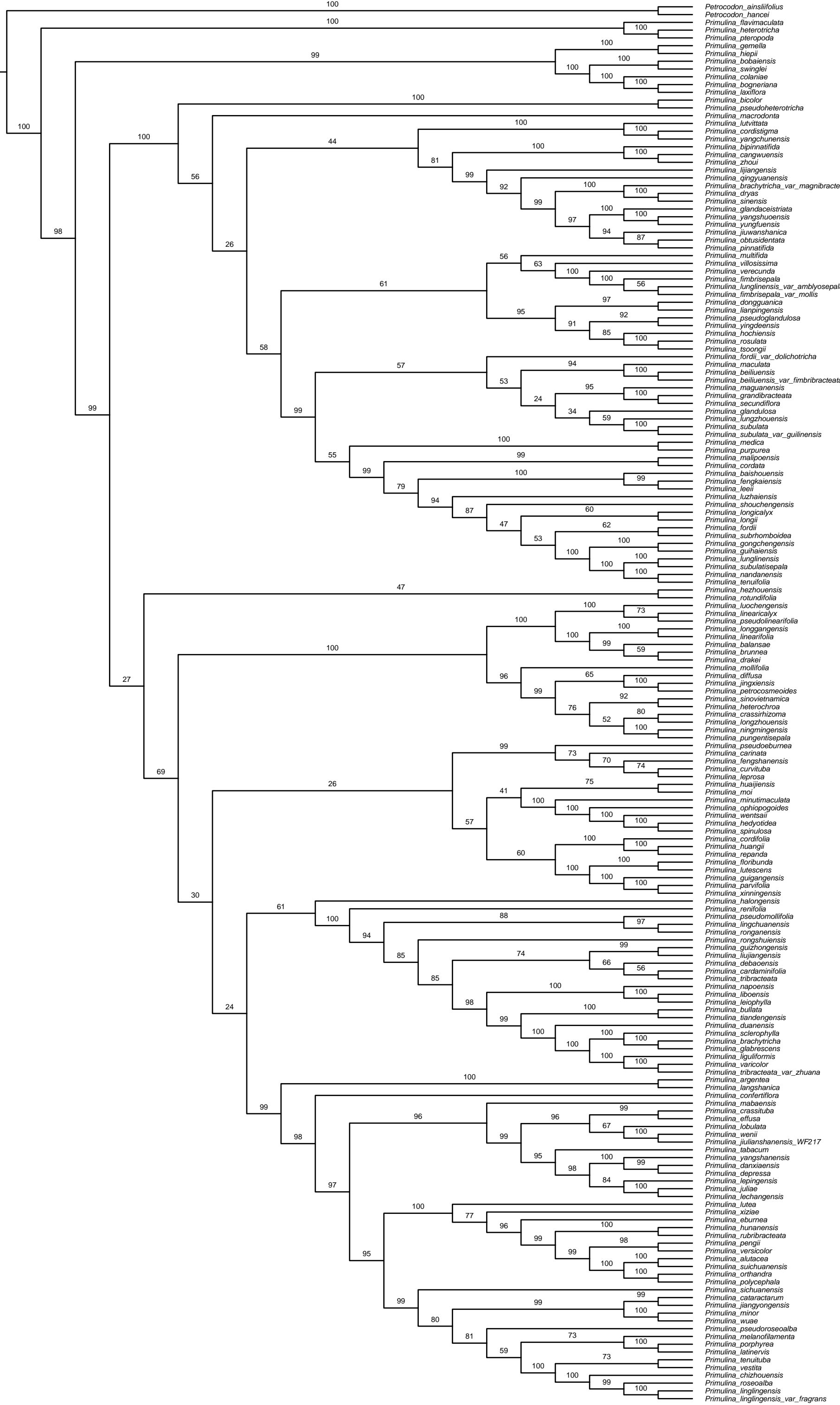

Supplement: Supplementary material 2 — The tree of the ITS nuclear marker for Primulina species [file phytokeys-226-001_article-96351__-s002.pdf]
